# The Stiffest and Strongest Predicted Material: C_2_N Atomic Chains Approach the Theoretical Limits

**DOI:** 10.1002/advs.202204884

**Published:** 2023-04-23

**Authors:** Enlai Gao, Hang Yang, Yongzhe Guo, Steven O. Nielsen, Ray H. Baughman

**Affiliations:** ^1^ Department of Engineering Mechanics Wuhan University Wuhan Hubei 430072 China; ^2^ Department of Chemistry and Biochemistry The University of Texas at Dallas Richardson TX 75080 USA; ^3^ Alan G. MacDiarmid NanoTech Institute The University of Texas at Dallas Richardson TX 75080 USA

**Keywords:** extreme mechanical properties, gravimetric modulus, gravimetric strength, linear atomic chains, theoretical limit

## Abstract

Though linear atomic chains exhibit extreme properties, it is presently unclear how these properties can be maximized by the choice of elemental composition. Considering that boron, carbon, and nitrogen can form high modulus and high strength atomic chains, here an algorithm is developed to construct 143 possible atomic chains of these elements with 6 or fewer atoms in the primitive cell and explore their stabilities and mechanical properties by first‐principles calculations. It is found that the gravimetric modulus (1032 GPa g^−1^ cm^3^) and strength (108 GPa g^−1^ cm^3^) of the C_2_N chain significantly exceed those of any known material, including the previously stiffest predicted material (C chain, 945 GPa g^−1^ cm^3^) and the previously strongest predicted material (BC chain, 92 GPa g^−1^ cm^3^), and also approach the theoretical limits of gravimetric modulus (1036 GPa g^−1^ cm^3^) and strength (130 GPa g^−1^ cm^3^). Mechanistic analyses demonstrate that the higher gravimetric modulus and strength of the C_2_N chain, compared with the C and BC chains, originate from its short, stiff chemical bonding and the abnormal decrease in bond length alternation under tension. The likely ease of fabrication and potential synthesis routes for C_2_N chains are discussed.

## Graphical abstract

The stiffest and strongest C_2_N atomic chain is discovered by using first‐principles calculations. These values of gravimetric modulus and strength approach the theoretical limits. Further analyses of the electronic and bonding structures show that the ultrahigh gravimetric modulus and strength are derived from the short, stiff C—C and C—N bonds and the abnormal decrease of bond length alternation under tension.

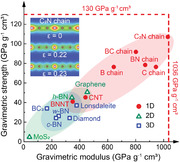

## 1 Introduction

Linear atomic chains exhibit extreme properties due to the anisotropy that results from the alignment of all chemical bonds along the chain axis.^[^ ^1^, ^2^, ^3^, ^4^, ^5^ ^]^ However, linear atomic chains usually have higher energy than bulk materials because of their low coordination, making their fabrication difficult and potentially dangerous.^[^ ^1^ ^]^ Fortunately, considerable effort has been devoted to the fabrication of atomic chains, such as the production of about 6000 atom‐long C chains inside double‐walled carbon nanotubes^[^ ^3^ ^]^ and deriving Au chains from gold nanocontacts,^[^ ^6^, ^7^ ^]^ C chains from graphene,^[^ ^8^ ^]^ P chains from black phosphorus,^[^ ^9^ ^]^ and BN chains from h‐BN.^[^ ^10^ ^]^

The modulus and strength are important for practical applications of linear atomic chains, such as assembling them into fibrous materials^[^ ^11^ ^]^ and constructing angstrom‐scale devices.^[^ ^7^, ^12^, ^13^ ^]^ For example, Gao et al.^[^ ^11^ ^]^ proposed a carbon assembly (nanotube‐wrapped C chains) with the highest Young's modulus of 1505 GPa among fibrous materials. However, the nominal cross‐sectional areas and therefore the mechanical properties of atomic chains are controversial.^[^ ^4^, ^14^, ^15^ ^]^ To avoid such controversy, we use gravimetric measures to characterize the mechanical properties of atomic chains. The gravimetric stress (*σ*_g_) is defined as the tensile stress of a sample (*σ*) divided by the mass density (*ρ*), which is equal to the tensile force divided by the linear mass density. Similar to gravimetric stress, gravimetric modulus and gravimetric strength are defined as the Young's modulus and tensile strength divided by the mass density, respectively. These metrics are independent of the cross‐sectional area for atomic chains.^[^ ^4^, ^11^, ^15^ ^]^ Herein, the gravimetric modulus and gravimetric strength are used as the metrics for modulus and strength, respectively. Materials previously predicted to possess the highest gravimetric modulus (C chain, 945 GPa g^−1^ cm^3^) and gravimetric strength (BC chain, 92 GPa g^−1^ cm^3^) are both linear atomic chains.^[^ ^4^ ^]^ For comparison, the theoretical limits of gravimetric modulus and strength for any material were previously predicted to be 1036 and 130 GPa g^−1^ cm^3^, respectively.^[^ ^16^ ^]^ This previous theoretical limit of gravimetric modulus was derived from the limits of the stiffness, alignment, and density of chemical bonds, while the theoretical limit of gravimetric strength was estimated from the theoretical limit of gravimetric modulus and the empirical modulus‐strength relation for defect‐free crystals (see details in ref. [16]).

According to Peierls theorem, one‐dimensional evenly spaced metallic chains will spontaneously undergo a transition into a more stable lower symmetry insulating state, resulting in the formation of alternating short strong and long weak bonds. However, Peierls distortions do not occur for all atomic chains. For example, lithium atomic chains do not undergo Peierls distortion, whereas carbon chains do.^[^ ^17^ ^]^ This distortion in C chains increases with increasing tensile strain,^[^ ^5^ ^]^ while B and BC chains do not exhibit Peierls distortions before their strains‐to‐failure.^[^ ^4^ ^]^ The increase of Peierls distortions in C chains under tensile strain results in premature breaking at a long bond. As a result, compared with C chains, B chains exhibit a higher gravimetric toughness and BC chains exhibit a higher gravimetric strength. This suggests that the discovery of atomic chains that do not undergo Peierls distortions might be a fruitful route to break the strength record. Furthermore, if the chemical bonds in a linear atomic chain are stiffer than those in C chains, this linear atomic chain might be stiffer. Hence, the discovery of atomic chains with chemical bonds stiffer than C chains and without Peierls instability is expected to break the modulus and strength records. Considering that light elements, such as B, C, and N, have the potential to form materials with high modulus and strength,^[^ ^4^, ^18^ ^]^ the family of atomic chains composed of these elements needs to be explored in more depth.

We here construct 143 possible atomic chains of combinations of boron, carbon, and nitrogen with 6 or fewer atoms in the primitive cell. Their stabilities and mechanical properties are analyzed by first‐principles calculations. Among these atomic chains, we show that the C_2_N chain has unparalleled properties. It is stable and has a gravimetric modulus (1032 GPa g^−1^ cm^3^) higher than the previously stiffest predicted material (C chain, 945 GPa g^−1^ cm^3^) and a gravimetric strength (108 GPa g^−1^ cm^3^) higher than the previously strongest predicted material (BC chain, 92 GPa g^−1^ cm^3^), both of which are close to the previously reported theoretical limits of gravimetric modulus (1036 GPa g^−1^ cm^3^) and strength (130 GPa g^−1^ cm^3^).^[^ ^16^ ^]^ Further analyses of the electronic and bonding structures show that the ultrahigh gravimetric modulus and strength are derived from the short, stiff C—C and C—N bonds and the abnormal decrease of bond length alternation under tension. Finally, possible fabrication methods for C_2_N chains are discussed.

## 2 Results and Discussion

### 2.1 Prescreening of Atomic Chains

**Figure 1:**
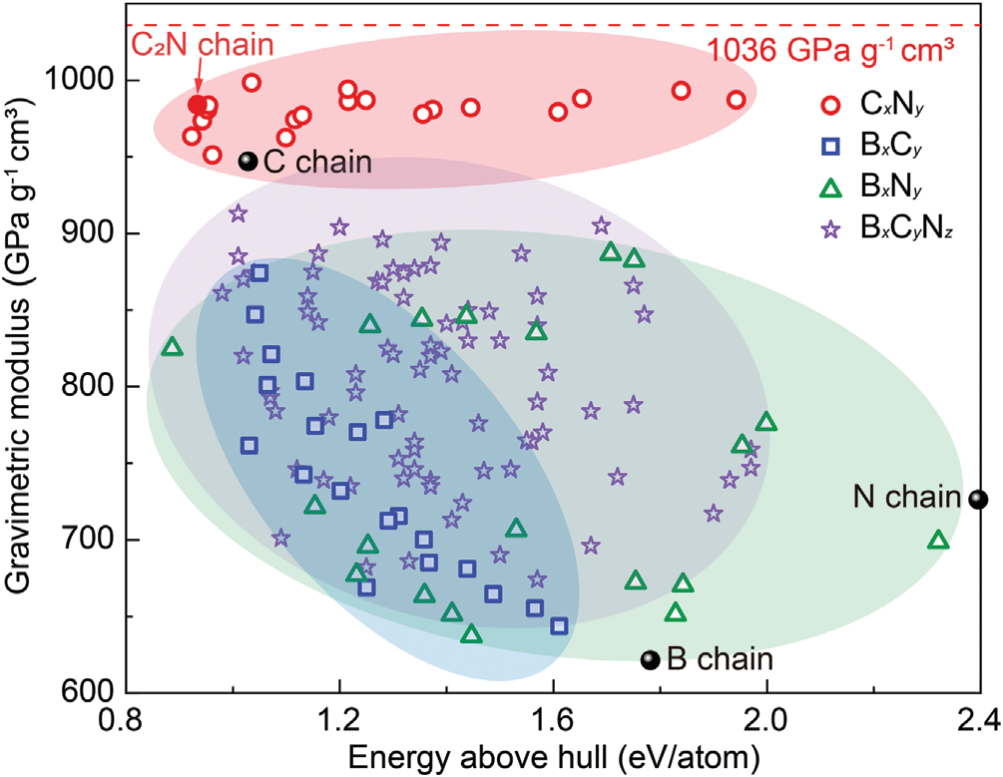
Screening for stable, high modulus atomic chains. Gravimetric moduli and energies above hull of linear atomic chains formed from B, C, and N elements. The dashed line indicates the theoretical limit of gravimetric modulus (1036 GPa g^−1^ cm^3^).^[^ ^16^ ^]^ These prescreening DFT calculations were conducted at the GGA functional level, while subsequent calculations used the more accurate HSE06 functional.

To identify the most promising candidates, we developed an algorithm to construct 143 possible atomic chains of combinations of boron, carbon, and nitrogen with 6 or fewer atoms in the primitive cell (see the Experimental Section and Table S1 in the Supporting Information for details), and calculated the energies above hull and gravimetric moduli for these chains from first principles. The energy above hull was calculated as the energy difference at zero pressure and zero temperature between the energy for an isolated linear chain and that of the lowest energy materials having the same overall chemical composition.^[^ ^4^ ^]^ For example, the energy above hull calculated for C*_x_* N*_y_* chains in this work is the same as the formation energy, which is referred to graphite and N_2_. The energy above hull relates to the ease of material synthesis, and a low energy above hull usually suggests easier synthesis than for a material having a high energy above hull. Considering a balance between accuracy and efficiency for this initial screening, first‐principles calculations at the GGA level were performed.^[^ ^19^ ^]^ The thereby calculated gravimetric modulus of the C chain (947 GPa g^−1^ cm^3^) is consistent with the value (982 GPa g^−1^ cm^3^) calculated using the literature‐reported linear mass density and force constants for this chain.^[^ ^20^ ^]^ The optimized structures of these 143 atomic chains are shown in Figure S1 (Supporting Information), and the gravimetric moduli and energies above hull are shown in **Figure** **1**.

Among the 143 atomic chains considered, C*_x_* N*_y_* chains possess generally high gravimetric moduli. The change of element ratio (*x* and *y*) seems to mainly affect the stability, while all C*_x_* N*_y_* chains show a higher gravimetric modulus than the C chain. These results indicate that C and N can form stiff chemical bonds, which impart chains of their combinations with ultrahigh moduli. Unfortunately, only a few C*_x_* N*_y_* chains were shown to be stable in the following investigation. B*_x_* C*_y_* chains have the most significant modulus dependence, since the modulus decreases as the boron fraction increases. Neither the upper nor the lower limits of their moduli escape from the range defined by monatomic C and B chains. B*_x_* N*_y_* atomic chains have the most varied performance. For the same atomic ratio, the proportion of B—N bonds in the chain almost directly determines performance: if there are more B—N bonds, the chains tend to be more stable and stiffer; otherwise, they are less stable and softer. An example of this is the large gap between –B–N– (gravimetric modulus of 825 GPa g^−1^ cm^3^ and energy above hull of 0.89 eV/atom) and –B–B–B–N–N–N– (gravimetric modulus of 699 GPa g^−1^ cm^3^ and energy above hull of 2.32 eV/atom) with the same atomic ratio. The number of atomic chains formed by ternary compounds B*_x_* C*_y_* N*_z_* reaches 80, more than the total number of binary and monatomic chains. However, the ternary combinations do not show high stiffness and their moduli are all lower than C*_x_* N*_y_*, which is attributed to the presence of boron. In general, we found that the presence of boron reduces the gravimetric modulus, whereas the presence of nitrogen increases the gravimetric modulus (Figure S2, Supporting Information).

### 2.2 Stabilities of High Modulus C*_x_* N*_y_* Chains

**Figure 2:**
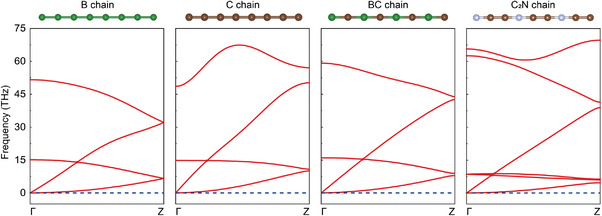
Phonon dispersion curves of the C_2_N chain and chains that previously held the record for gravimetric modulus (C chain), strength (BC chain), and toughness (B chain).^[^ ^4^ ^]^

The prescreening process showed that 20 C*_x_* N*_y_* chains are the most promising candidates. Therefore, we further examined these chains. First, phonon calculations were used to check the stability of these chains. These calculations show that 4 (C_2_N, C_3_N, C_4_N, and C_5_N) of the 20 C*_x_* N*_y_* chains do not have imaginary frequencies (**Figure** **2**; Figure S3, Supporting Information), indicating that these chains are stable. Considering that the C_2_N chain has the highest gravimetric modulus, we further studied its properties. In fact, the phonon stability of the C_2_N chain has been demonstrated at 0 K. To investigate the stability of the C_2_N chain at a higher temperature, we conducted ab initio molecular dynamics (AIMD) simulations. An Andersen thermostat was used to control the temperature.^[^ ^21^ ^]^ The 12‐atom C_2_N chain supercell upheld its structural integrity during the 1 ns AIMD simulation at 300 K (Movie S1, Supporting Information). These results indicate the metastability of the isolated C_2_N chain at room temperature. Additionally, the stability of linear atomic chains with respect to inter‐chain reactions is important.^[^ ^1^, ^22^, ^23^ ^]^ Guided by the observed formation of four‐membered rings for nonstretched C chains,^[^ ^5^, ^24^ ^]^ we modeled such an inter‐chain reaction for nonstretched C_2_N chains. Figure S4 (Supporting Information) shows the energy profile for this reaction, which assumes that the cross‐linking reaction is not prohibited by a high applied tensile strain. Compared with the kinetic barriers for these inter‐chain reactions of C (1.9 eV) and BC chains (0.5 eV),^[^ ^4^ ^]^ the kinetic barrier of the C_2_N chain is substantial (1.3 eV), indicating the hindrance of this reaction even in the absence of an applied tensile strain.

### 2.3 Mechanical Properties of the C_2_N Chain

**Figure 3:**
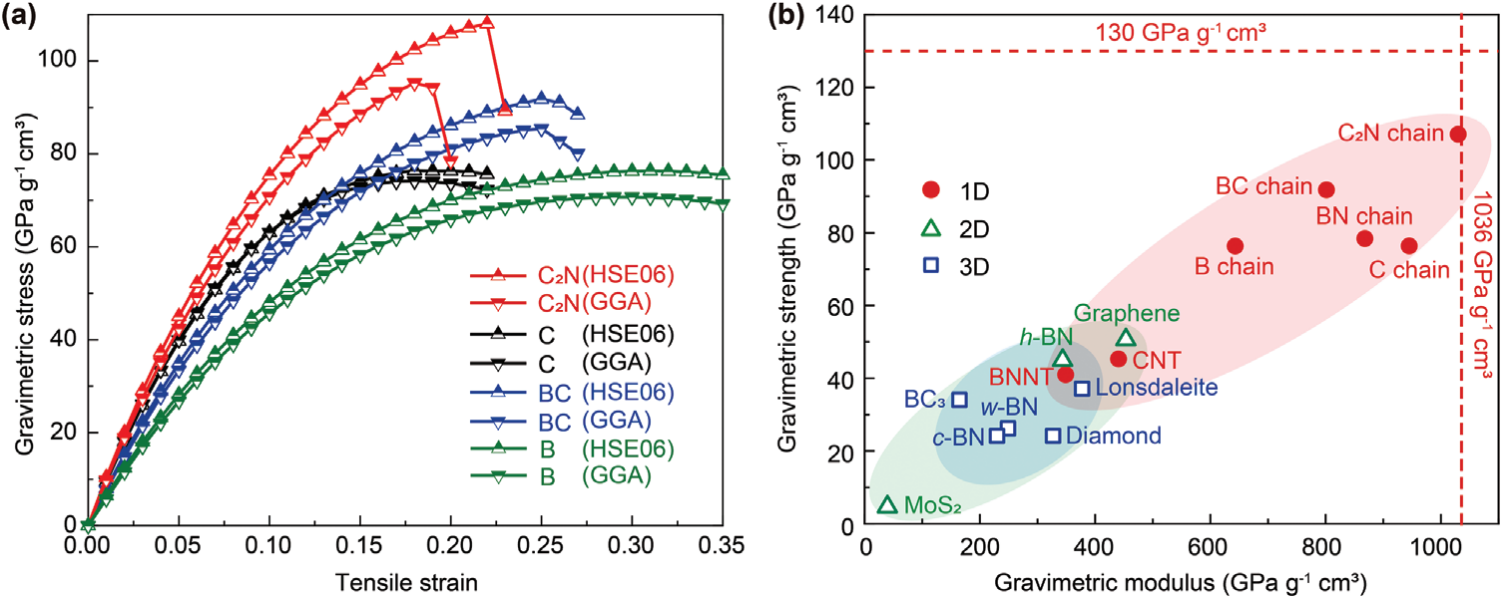
Mechanical properties of the C_2_N chain compared with other high‐performance materials. a) DFT calculations of gravimetric stress–strain curves for B, C, BC, and C_2_N chains at the GGA and HSE06 functional levels. b) Gravimetric strength and modulus of the C_2_N chain compared to other high‐performance materials tabulated in ref.[4] The dashed lines indicate the theoretical limits determined in our previous work.^[^ ^16^ ^]^

To verify the mechanical properties of the newly discovered stable high‐modulus C_2_N chain, we conducted tensile tests using a more accurate DFT method (**Figure** **3**a). These DFT calculations were conducted using the hybrid HSE06 functional. The tensile tests show that the C_2_N chain has unparalleled properties. We found that the C_2_N chain has a higher gravimetric toughness (15.7 kJ g^−1^) than any other material except the B chain (16.9 kJ g^−1^).^[^ ^4^ ^]^ The gravimetric modulus of the C_2_N chain (1032 GPa g^−1^ cm^3^) exceeds the C chain (945 GPa g^−1^ cm^3^) and breaks the record of gravimetric modulus to become the stiffest predicted material, which is extremely close to the theoretical limit determined in our previous work (1036 GPa g^−1^ cm^3^; Figure 3b).^[^ ^16^ ^]^ Equally interestingly, we found that the gravimetric strength of the C_2_N chain (108 GPa g^−1^ cm^3^) is significantly higher than the C chain (76 GPa g^−1^ cm^3^), and even exceeds the BC chain (92 GPa g^−1^ cm^3^), which was previously predicted to be the strongest material (Figure 3b). These calculations indicate that the C_2_N chain not only breaks the records, but also approaches the theoretical limits of gravimetric modulus and strength. To our knowledge, the gravimetric modulus and strength of the C_2_N chain are higher than for any other material, and therefore this chain is the stiffest and strongest predicted material.

The above‐reported mechanical properties were investigated at 0 K. Previous work demonstrated the failure behaviors of the linear C chain including temperature effects,^[^ ^5^ ^]^ indicating that this chain can well maintain its strength at 300 K or above. We here further investigated the failure behaviors of the C_2_N chain including temperature effects (see Note S1 and Figure S5 in the Supporting Information for details). We found that the average strength of the C_2_N chain at 300 K for 1 day is 88% of the strength at 0 K, indicating that the C_2_N chain would not break up when small strains are applied at room temperature (300 K). To provide additional support, we did AIMD simulations for a C_2_N chain with a length of about 1.5 nm under various tensile strains (0%, 3%, 6%, 9%, and 12%). An Andersen thermostat was used to control the temperature at 300 K.^[^ ^21^ ^]^ For the duration of the simulations (1 ns), the structures under tensile strains of 0%, 3%, 6%, and 9% maintain their structural integrity, and only the structure under a tensile strain of 12% breaks at 11.6 ps (Movie S1, Supporting Information). These results demonstrate the considerable strength of C_2_N chains at room temperature. Additionally, we evaluated the mechanical properties of finite C_2_N chains (3–18 atoms) as compared with the above‐reported mechanical properties of the infinite C_2_N chain. The calculated gravimetric moduli for finite C_2_N chains of length over 12 atoms are greater than 90% of that of the infinite C_2_N chain (Figure S6, Supporting Information).

To explore bending behaviors, we calculated the bending stiffnesses of the atomic chains. The bending stiffness (*k*_b_) was calculated from the energy (*E*_b_) required to bend a sufficiently long atomic chain into a circle with a radius *R*: *k*_b_ = *E*_b_ *R*/*π*.^[^ ^4^, ^5^ ^]^ The thereby calculated bending stiffnesses for linear B, C, BC, and C_2_N chains are 1.7, 3.7, 1.8, and 3.3 eV Å, respectively. From these bending stiffnesses, the C_2_N chain is slightly easier to bend than the C chain, and more difficult to bend than the B and BC chains.

### 2.4 Mechanism for the Ultrahigh Modulus and Strength of the C_2_N Chain

**Figure 4:**
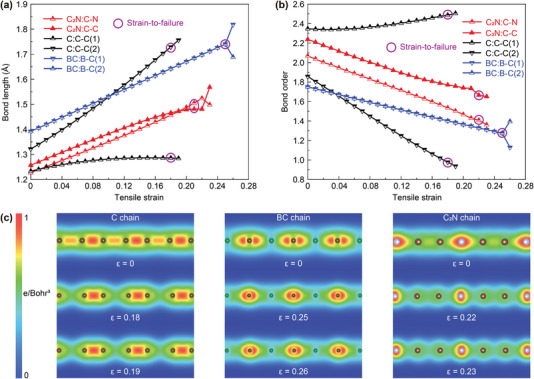
Electronic and bonding structures of the C, BC, and C_2_N chains. a) Bond length and b) bond order of the C, BC, and C_2_N chains as a function of tensile strain. The strains‐to‐failure are marked with purple circles. The bond length and order alternations are defined as the absolute difference of bond lengths and orders for the long and short bonds, respectively. c) Charge density distribution for the C, BC, and C_2_N chains under 0% strain, strain‐to‐failure, and a strain 1% higher than the strain‐to‐failure.

Since the C chain was previously predicted to have the highest gravimetric modulus, and the BC chain was previously predicted to have the highest gravimetric strength, in this section, we compared these properties to those of the C_2_N chain. Because the gravimetric modulus is determined for small strains, we here compared the bond lengths and bond orders for the unstrained chains. The lengths of the long and short bonds in the C_2_N chain are smaller than the corresponding bonds in the C chain, resulting in the average bond length of the C_2_N chain being smaller than that of the C chain (**Figure** **4**a). Meanwhile, the average bond order of the C_2_N chain is larger than that of the C chain (Figure 4b). More importantly, the bond alternation of the C_2_N chain (bond length alternation of 0.03 Å and bond order alternation of 0.18) is much smaller than for the C chain (bond length alternation of 0.09 Å and bond order alternation of 0.49). Compared with the previously stiffest predicted material (C chain), the shorter average bond length, larger average bond order, and smaller bond alternation of the C_2_N chain account for its higher gravimetric modulus.

Since the gravimetric strength is determined by structures near the strain‐to‐failure, we here compared the bond lengths and orders of chains near the strain‐to‐failure. Both the lengths of the long and short bonds in the C_2_N chain near the strain‐to‐failure are smaller than those of the BC chain (Figure 4a). Meanwhile, both the bond orders of the long and short bonds in the C_2_N chain near the strain‐to‐failure are larger than those of the BC chain (Figure 4b). More importantly, the bond length alternation of the C_2_N chain decreases as the tensile strain increases. The electron densities further support the strain dependence of bonding structures for the C, BC, and C_2_N chains under tension (Figure 4c). Compared to the previously strongest predicted material (BC chain), the shorter bond lengths and larger bond orders near the strain‐to‐failure, and the strain‐induced decrease of bond length alternation of the C_2_N chain account for its higher gravimetric strength.

### 2.5 Likely Ease of Fabrication and Possible Synthesis of the C_2_N Chain

The energy above hull^[^ ^4^ ^]^ is used to characterize the formability and stability of atomic chains. Our calculations show that the energies above hull of B, C, BC, and C_2_N chains are 1.77, 1.03, 1.31, and 0.93 eV /atom, respectively, and therefore among these chains, the C_2_N chain shows the lowest energy above hull. The energies above hull of four stable C*_x_* N*_y_* chains (C_2_N, C_3_N, C_4_N, and C_5_N) compared with the C chain also show that the C_2_N chain has the lowest energy above hull, indicating that the C_2_N chain is the most stable (Figure S7, Supporting Information). This situation is quite unusual, since normally the high‐modulus, high‐strength linear chain materials have a high energy above hull. For example, the linear C chain is even potentially explosive.^[^ ^1^ ^]^ In summary, these results generally suggest the most likely ease of fabrication of the C_2_N chain compared with other high‐performance atomic chains.

Previous studies have shown that C chains can be derived from graphene,^[^ ^8^ ^]^ BN chains from h‐BN,^[^ ^10^ ^]^ and P chains from few‐layer black phosphorus.^[^ ^9^ ^]^ These results suggest that a possible way to obtain linear atomic chains is to produce them from two‐dimensional phases with the same chemical compositions, element ratios, and building blocks. We found that there are two literature reports of C_2_N sheets containing (C_2_N)*_n_* building blocks, e.g., holey C_2_N sheets^[^ ^25^, ^26^, ^27^ ^]^ and nonholey (or graphene‐like) C_2_N sheets.^[^ ^28^ ^]^ However, only holey C_2_N sheets have been synthesized.^[^ ^25^, ^26^, ^27^ ^]^ Considering these literature results, we conducted simulations of drawing holey C_2_N and nonholey C_2_N sheets (see Note S2, Figure S8, and Movies S2 and S3 in the Supporting Information for details). Similar to previous simulations of drawing C chains from a graphene ribbon,^[^ ^29^ ^]^ C_2_N chains or segments were observed in these processes. More specifically, about ten‐atom‐long C_2_N chains were observed during the drawing of nonholey C_2_N sheets and holey C_2_N sheets. It might be possible to increase the length of this C_2_N chain by optimizing the drawing process, for example by preferentially heating the sheets so that the chain separation from the sheets is preferred over chain rupture. On the other hand, the fabrication of C_2_N chains within nanotubes might also be possible. This motivation is provided by the fact that linear C chains have been produced by heating double‐ and multi‐wall carbon nanotubes having a small diameter for the innermost nanotube.^[^ ^3^, ^30^ ^]^ This nanotube confinement can stabilize the produced linear C chain. Like the case for linear C chains, if double‐ or multi‐wall C_2_N nanotubes can be fabricated in the future, it might be possible to fabricate linear C_2_N chains in an analogous way. Additionally, many stabilization methods have been developed, such as using a carbon nanotube sheath as a confining host^[^ ^3^, ^30^, ^31^ ^]^ and using end‐capping functional groups for isolation.^[^ ^32^, ^33^, ^34^ ^]^

## 3 Conclusion

We predict the atomic chain properties of combinations of boron, carbon, and nitrogen limited to 6 or fewer atoms in the primitive cell. In general, we find that the presence of boron reduces the gravimetric modulus, whereas the presence of nitrogen increases the gravimetric modulus. Although we initially thought that the highest performance materials should be free of a Peierls distortion, another possibility arose during our investigation, namely that the bond length alternation could decrease under applied tensile strain. Such a situation occurs for the C_2_N chain, whose mechanical, dynamical, and thermodynamic stabilities are established, and whose gravimetric modulus and strength are both close to the theoretical limits. The gravimetric modulus of the C_2_N chain (1032 GPa g^−1^ cm^3^) exceeds that of the previously predicted stiffest atomic chain (945 GPa g^−1^ cm^3^ for the C chain), and the gravimetric strength (108 GPa g^−1^ cm^3^) exceeds that of the previously predicted strongest atomic chain (92 GPa g^−1^ cm^3^ for the BC chain). Compared to the C chain, the shorter average bond length, larger average bond order, and smaller bond order and length alternations of the C_2_N chain account for its higher gravimetric modulus. Compared to the BC chain, the shorter bond length and larger bond order near the strain‐to‐failure, and the strain‐induced decrease in bond length alternation of the C_2_N chain account for its higher gravimetric strength. Finally, the likely ease of fabrication and potential synthesis of the C_2_N chain from the known C_2_N sheet structures are discussed.

## 4 Experimental Section

### Constructions of all Possible Linear Atomic Chains

All atomic chains were generated based on the constraint that the primitive cell contains 6 or fewer atoms and consists of combinations of boron, carbon, and nitrogen. The strategy to produce these chains includes three steps. First, the number of possible atomic chains is counted by the Pólya Enumeration Theorem (PET).^[^ ^35^ ^]^ This results in 143 unique atomic chains. Secondly, we enumerate the arrangement of the 143 atomic chains (Table S1, Supporting Information), and the bond lengths before DFT optimization were set at 90% of the sum of the covalent radii of the neighboring atoms.^[^ ^36^ ^]^ Finally, DFT optimizations were conducted on these atomic chains for energy‐minimized structures.

The key point of this strategy is to ensure that enumerations are not duplicated or omitted. We rely on the mature PET method for counting equivalence classes. One typical application of the PET method is for enumerating the number of strings consisting of *n* beads with *m* different colors, which is called *necklace* enumeration. Herein, the counting of atomic chains considering the periodic nature of the primitive cell is a *dihedral necklace* enumeration. Using PET, we can calculate the number of atomic chains for a given number of atoms in the primitive cell.^[^ ^35^, ^37^ ^]^ Specifically, for atomic chains consisting of *n* atoms with *m* elements, the number *N* of unique atomic chains is

(1) $$N=\frac{1}{2n}\sum_{d|n}\varphi \left(d\right)m^{\frac{n}{d}}+\left\{\begin{array}{cc} \frac{m+1}{4}\left(m^{\frac{n}{2}}\right) & n=2x,x\in N^{*}\\ \frac{1}{2}m^{\frac{n+1}{2}} & n=2x+1,x\in N^{*} \end{array}\right.$$

where *d* is a factor of *n* (including *n*) and the Euler function *φ*(*d*) is the number of positive integers less than *d* that are coprime to *d*, which can be calculated by

(2) $$\varphi \left(d\right)=d\prod \left(1-\frac{1}{p_{j}}\right)$$

where *p_j_* is a prime number, *gcd*(*d*, *p_j_*) > 1, and *φ*(1) = 1. The total number of atomic chains with 1, 2, 3, 4, 5, and 6 atoms in the primitive cell are 3, 6, 10, 21, 39, and 92, respectively. By deleting 28 duplicates (e.g., –B– and –B–B– for 1 and 2 atoms in the primitive cells), we obtained a total of 143 unique atomic chains. Notably, considering the Peierls instability, a primitive cell containing 2 atoms was adopted for monatomic chains (Table S1, Supporting Information).

### First‐Principles Calculations

To investigate the structures and properties of atomic chains, we performed first‐principles calculations based on the density functional theory (DFT) framework using the Vienna Ab‐Initio Simulation Package (VASP).^[^ ^38^ ^]^ Unless otherwise noted, the Perdew–Burke–Ernzerhof parameterization^[^ ^19^ ^]^ of the generalized gradient approximation (GGA) was used for the exchange‐correlation functional. To verify the mechanical properties of the newly discovered stable high‐modulus C_2_N chain, we conducted tensile tests using DFT calculations with the hybrid HSE06 functional that is more reliable for calculating band structures and bond alternation than the standard GGA^[^ ^5^, ^39^, ^40^ ^]^ (Figure 3a). The bond length alternation (BLA) caused by Peierls instability under tension is sensitive to the electronic exchange interaction, which is poorly described by regular density functional theory, making the use of hybrid functionals desirable.^[^ ^40^ ^]^ In the hybrid HSE06 functional, larger amounts of exact exchange can lead to wider band gaps and stronger electron–phonon coupling. This more accurate HSE06 functional yields an increase of BLA from 0.088 Å in strain‐free carbyne to 0.248 Å when the strain increases to 10%,^[^ ^5^ ^]^ while the GGA functional underestimates the BLA.^[^ ^5^ ^]^ Considering that the accuracy of BLA is important to the gravimetric strength of atomic chains, our DFT calculations for tensile properties were conducted using the hybrid HSE06 functional. Projector augmented wave potentials were employed to treat interactions between ions and electrons.^[^ ^41^ ^]^ We used an energy cutoff of 1.3 times the default value and an ≈75 *k*‐point mesh along the chain axis for Brillouin zone sampling.^[^ ^42^ ^]^ All atomic positions and axial lattice parameters of the structures were optimized until all forces were below 0.01 eV/Å. To minimize the interaction between periodic images, a vacuum layer of 30 Å was used.

To investigate the behavior of the C_2_N chain at room temperature on the nanosecond time scale, a *k*‐point mesh with a density of about 30 Å was used in AIMD simulations. These AIMD simulations on the nanosecond time scale were accelerated by the on‐the‐fly machine learning force field generation method^[^ ^43^, ^44^ ^]^ incorporated in the VASP code. An Andersen thermostat was used to control the temperature at 300 K.^[^ ^21^ ^]^

To measure the Peierls distortion, the bond length alternation and the bond order alternation of linear atomic chains were used as criteria, in which the bond order is calculated based on a refinement of the density derived electrostatic and chemical approach.^[^ ^45^ ^]^ We explored the effect of spin polarization in the DFT calculations (Figure S9, Supporting Information). Gravimetric stress–strain curves for the C_2_N chain show the negligible effect of spin polarization. Hence, spin polarization was not included in the DFT calculations. We additionally did mechanical tests for the C_2_N chain with 3, 6, 9, 12, and 15 atoms in the computational cell. According to our calculations, the mechanical properties of [–C–C–N–]*_n_* chains are almost identical (Figure S10, Supporting Information) as *n* increases from 1 to 5, indicating that the number of atoms in the smallest computational cell is sufficient for the convergence of the models.

## Conflict of Interest

The authors declare no conflict of interest.

## Supporting information

Supporting Information

Supplemental Movie 1

Supplemental Movie 2

Supplemental Movie 3

## Data Availability

The data that support the findings of this study are available from the corresponding author upon reasonable request.
