# Supplementary material for: The Stiffest and Strongest Predicted Material: C2N Atomic Chains Approach the Theoretical Limits
Source: Adv Sci (Weinh). 2023 Apr 23;10(20):2204884. doi: 10.1002/advs.202204884 (PMC10369241; doi:10.1002/advs.202204884)
Supplement: Supplementary file 1 — Supporting Information [file ADVS-10-2204884-s004.pdf]

ADVANCED SCIENCE
Open Access

Supporting Information

for *Adv. Sci.*, DOI 10.1002/advs.202204884

The Stiffest and Strongest Predicted Material: C_2N Atomic Chains Approach the Theoretical Limits

Enlai Gao, Hang Yang, Yongzhe Guo, Steven O. Nielsen* and Ray H. Baughman**

Supporting Information

The Stiffest and Strongest Predicted Material: C_2N Atomic Chains Approach the Theoretical Limits

Enlai Gao, Hang Yang, Yongzhe Guo, Steven O. Nielsen*, and Ray H. Baughman**

Enlai Gao, Hang Yang, Yongzhe Guo

Department of Engineering Mechanics, Wuhan University, Wuhan, Hubei 430072, China.

Email: enlaigao@whu.edu.cn (E.G.)

Steven O. Nielsen, Ray H. Baughman

Department of Chemistry and Biochemistry, The University of Texas at Dallas, Richardson, TX 75080, USA.

Emails: steven.nielsen@utdallas.edu (S.O.N.); ray.baughman@utdallas.edu (R.H.B.)

Ray H. Baughman

Alan G. MacDiarmid NanoTech Institute, The University of Texas at Dallas, Richardson, TX 75080, USA.

Note 1: Failure behaviors including temperature effects

We investigated the effect of temperatures up to 500 K on the tensile strength of C_2N chains. At a finite temperature, the average failure rate for a single bond follows the Arrhenius form:[1,2]

$$R(\sigma) = v(f)e^{-\Delta E(\sigma)/k_B T}, \tag{1}$$

where $v(f)$ is approximately 10^{13} Hz (the "attempt rate" to cross over the energy barrier), $\Delta E(\sigma)$ is the energy barrier to break a single bond, and k_B and T are the Boltzmann constant and temperature, respectively. From **Eq. (1)** and the DFT calculated energy barrier $\Delta E(\sigma)$ for the C_2N chain, we predicted the relative strength (averaged strength at a finite temperature divided by the strength at 0 K) of the C_2N chain for a duration of 1 day as a function of temperature (Figure S5).

Note 2: Simulation details of drawing C_2N chains from C_2N sheets

Previous experimental production of C chains and BN chains was made by removing atoms row by row from graphene and BN sheets through electron irradiation,[3] and chain structures were formed from the thereby produced narrow ribbon structures. The drawing of C chains from a graphene ribbon has also been simulated.[4] Similar to previous simulations, we constructed initial narrow ribbon structures of holey C_2N and non-holey C_2N sheets (Figure S8). After structural optimization, we gradually stretched these structures until rupture by applying strain increments of 1% and minimizing the energy after each increment (Movies S2 and S3). Similar to previous simulations of drawing C chains from a graphene ribbon,[4] we found C_2N chains or segments during the drawing of non-holey C_2N sheets and holey C_2N sheets (Figure S8). Considering the balance between computational accuracy and efficiency, these simulations were performed by DFTB+ program.[5] For the third-order variant of DFTB, the 3ob parametrization is employed.[6]

Supporting Table

Table S1. Chemical structures, gravimetric moduli (GGA), energies above hull, and stability of 143 unique atomic chains.

Index	Chemical formulas	Bonding topologies	Gravimetric moduli ($GPa{\cdot}g^{-1}{\cdot}cm^3$)	Energies above hull (eV/atom)	Dynamical stability
1	B_2	-B-B-	621	1.78	√
2	C_2	-C-C-	941	1.03	√
3	N_2	-N-N-	726	2.40	×
4	BC	-B-C-	778	1.28	√
5	B_2C	-B-C-B-	669	1.25	-
6	BC_2	-C-B-C-	801	1.07	-
7	B_3C	-B-B-C-B-	665	1.49	-
8	BC_3	-C-C-B-C-	821	1.07	-
9	B_2C_2	-B-B-C-C-	770	1.23	-
10	B_4C	-B-B-B-C-B-	655	1.57	-
11	BC_4	-C-C-C-B-C-	847	1.04	-
12	B_3C_2	-B-B-B-C-C-	700	1.36	-
13	B_3C_2	-B-B-C-B-C-	715	1.31	-
14	B_2C_3	-C-C-C-B-B-	743	1.13	-
15	B_2C_3	-C-C-B-C-B-	762	1.03	-
16	B_5C	-B-B-B-B-C-B-	644	1.61	-
17	BC_5	-C-C-C-C-B-C-	874	1.05	-
18	B_4C_2	-B-B-B-B-C-C-	681	1.44	-
19	B_4C_2	-B-B-B-C-B-C-	685	1.37	-
20	B_2C_4	-C-C-C-C-B-B-	774	1.15	-
21	B_2C_4	-C-C-C-B-C-B-	803	1.14	-
22	B_3C_3	-B-B-B-C-C-C-	712	1.29	-
23	B_3C_3	-B-B-C-B-C-C-	732	1.20	-
24	BN	-B-N-	825	0.89	-
25	B_2N	-B-N-B-	677	1.23	-
26	BN_2	-N-B-N-	844	1.35	-
27	B_3N	-B-B-N-B-	664	1.36	-
28	BN_3	-N-N-B-N-	835	1.57	-

29	B_2N_2	-B-B-N-N-	672	1.75	-
30	B_4N	-B-B-B-N-B-	651	1.41	-
31	BN_4	-N-N-N-B-N-	887	1.71	-
32	B_3N_2	-B-B-B-N-N-	670	1.84	-
33	B_3N_2	-B-B-N-B-N-	722	1.15	-
34	B_2N_3	-N-N-N-B-B-	761	1.95	-
35	B_2N_3	-N-N-B-N-B-	840	1.26	-
36	B_5N	-B-B-B-B-N-B-	637	1.45	-
37	BN_5	-N-N-N-N-B-N-	883	1.75	-
38	B_4N_2	-B-B-B-B-N-N-	651	1.83	-
39	B_4N_2	-B-B-B-N-B-N-	696	1.25	-
40	B_2N_4	-N-N-N-N-B-B-	776	2.00	-
41	B_2N_4	-N-N-N-B-N-B-	846	1.44	-
42	B_3N_3	-B-B-B-N-N-N-	699	2.32	-
43	B_3N_3	-B-B-N-B-N-N-	706	1.53	-
44	CN	-C-N-	998	1.04	×
45	C_2N	-C-N-C-	984	0.93	√
46	CN_2	-N-C-N-	987	1.25	×
47	C_3N	-C-C-N-C-	968	0.94	√
48	CN_3	-N-N-C-N-	988	1.65	×
49	C_2N_2	-C-C-N-N-	986	1.22	×
50	C_4N	-C-C-C-N-C-	966	0.95	√
51	CN_4	-N-N-N-C-N-	993	1.84	×
52	C_3N_2	-C-C-C-N-N-	963	1.10	×
53	C_3N_2	-C-C-N-C-N-	963	0.92	×
54	C_2N_3	-N-N-N-C-C-	982	1.45	×
55	C_2N_3	-N-N-C-N-C-	994	1.22	×
56	C_5N	-C-C-C-C-N-C-	951	0.96	√
57	CN_5	-N-N-N-N-C-N-	987	1.94	×
58	C_4N_2	-C-C-C-C-N-N-	974	1.12	×
59	C_4N_2	-C-C-C-N-C-N-	984	0.95	×
60	C_2N_4	-N-N-N-N-C-C-	979	1.61	×
61	C_2N_4	-N-N-N-C-N-C-	981	1.37	×
62	C_3N_3	-C-C-C-N-N-N-	978	1.36	×

63	C_3N_3	-C-C-N-C-N-N-	977	1.13	×
64	BCN	-B-C-N-	850	1.44	-
65	B_2CN	-B-B-C-N-	724	1.43	-
66	B_2CN	-B-C-B-N-	780	1.18	-
67	BC_2N	-C-C-N-B-	842	1.16	-
68	BC_2N	-C-B-C-N-	830	1.50	-
69	BCN_2	-N-N-B-C-	830	1.44	-
70	BCN_2	-N-B-N-C-	870	1.02	-
71	B_2C_2N	-B-B-C-C-N-	753	1.31	-
72	B_2C_2N	-B-B-C-N-C-	746	1.52	-
73	B_2C_2N	-B-N-B-C-C-	793	1.07	-
74	B_2C_2N	-B-C-B-C-N-	782	1.31	-
75	B_2CN_2	-B-B-C-N-N-	739	1.93	-
76	B_2CN_2	-B-B-N-C-N-	764	1.56	-
77	B_2CN_2	-B-C-B-N-N-	788	1.75	-
78	B_2CN_2	-B-N-B-C-N-	811	1.35	-
79	BC_2N_2	-C-C-N-N-B-	879	1.37	-
80	BC_2N_2	-C-C-N-B-N-	913	1.01	-
81	BC_2N_2	-C-B-C-N-N-	859	1.57	-
82	BC_2N_2	-C-N-C-N-B-	904	1.20	-
83	B_3CN	-B-B-B-C-N-	690	1.50	-
84	B_3CN	-B-B-C-B-N-	701	1.09	-
85	BC_3N	-C-C-C-N-B-	869	1.27	-
86	BC_3N	-C-C-N-C-B-	842	1.43	-
87	BCN_3	-N-N-N-B-C-	840	1.57	-
88	BCN_3	-N-N-B-N-C-	885	1.01	-
89	$B_2C_2N_2$	-B-B-C-C-N-N-	741	1.72	-
90	$B_2C_2N_2$	-B-B-C-N-C-N-	809	1.59	-
91	$B_2C_2N_2$	-B-B-N-C-C-N-	823	1.39	-
92	$B_2C_2N_2$	-B-N-B-C-N-C-	759	1.34	-
93	$B_2C_2N_2$	-B-N-B-N-C-C-	784	1.08	-
94	$B_2C_2N_2$	-B-B-C-N-N-C-	759	1.97	-
95	$B_2C_2N_2$	-B-N-N-B-C-C-	745	1.47	-
96	$B_2C_2N_2$	-B-C-B-N-C-N-	825	1.29	-

97	$B_2C_2N_2$	-B-C-N-B-N-C-	841	1.40	-
98	$B_2C_2N_2$	-B-C-B-N-N-C-	784	1.67	-
99	B_3C_2N	-B-B-B-C-C-N-	713	1.41	-
100	B_3C_2N	-B-B-B-C-N-C-	770	1.58	-
101	B_3C_2N	-B-N-B-B-C-C-	739	1.17	-
102	B_3C_2N	-B-B-C-B-C-N-	739	1.37	-
103	B_3C_2N	-B-B-C-B-N-C-	735	1.37	-
104	B_3C_2N	-B-N-B-C-B-C-	735	1.22	-
105	B_3CN_2	-B-B-B-C-N-N-	717	1.90	-
106	B_3CN_2	-B-B-B-N-C-N-	790	1.57	-
107	B_3CN_2	-B-C-B-B-N-N-	696	1.67	-
108	B_3CN_2	-B-B-N-B-C-N-	746	1.34	-
109	B_3CN_2	-B-B-C-N-B-N-	740	1.32	-
110	B_3CN_2	-B-C-B-N-B-N-	746	1.12	-
111	B_2C_3N	-C-C-C-B-B-N-	821	1.30	-
112	B_2C_3N	-C-C-C-B-N-B-	797	1.07	-
113	B_2C_3N	-C-C-N-C-B-B-	776	1.46	-
114	B_2C_3N	-C-C-B-C-N-B-	808	1.23	-
115	B_2C_3N	-C-C-N-B-C-B-	796	1.23	-
116	B_2C_3N	-C-N-C-B-C-B-	808	1.41	-
117	BC_3N_2	-C-C-C-N-N-B-	858	1.32	-
118	BC_3N_2	-C-C-C-N-B-N-	861	0.98	-
119	BC_3N_2	-C-B-C-C-N-N-	849	1.48	-
120	BC_3N_2	-C-C-N-C-N-B-	849	1.14	-
121	BC_3N_2	-C-C-N-B-C-N-	875	1.15	-
122	BC_3N_2	-C-B-C-N-C-N-	877	1.34	-
123	BC_2N_3	-N-N-N-C-C-B-	887	1.54	-
124	BC_2N_3	-N-N-N-C-B-C-	905	1.69	-
125	BC_2N_3	-N-N-B-N-C-C-	887	1.16	-
126	BC_2N_3	-N-N-C-N-B-C-	874	1.32	-
127	BC_2N_3	-N-N-B-C-N-C-	877	1.30	-
128	BC_2N_3	-N-B-N-C-N-C-	874	1.04	-
129	B_2CN_3	-N-N-N-B-B-C-	747	1.97	-
130	B_2CN_3	-N-N-N-B-C-B-	847	1.77	-

131	B_2CN_3	-N-C-N-N-B-B-	765	1.55	-
132	B_2CN_3	-N-N-B-N-B-C-	764	1.34	-
133	B_2CN_3	-N-N-B-N-C-B-	820	1.37	-
134	B_2CN_3	-N-C-N-B-N-B-	820	1.02	-
135	B_4CN	-B-B-B-B-C-N-	674	1.57	-
136	B_4CN	-B-B-B-C-B-N-	686	1.33	-
137	B_4CN	-B-B-C-B-B-N-	682	1.25	-
138	BC_4N	-C-C-C-C-B-N-	859	1.14	-
139	BC_4N	-C-C-C-B-C-N-	827	1.37	-
140	BC_4N	-C-C-B-C-C-N-	868	1.28	-
141	BCN_4	-N-N-N-N-B-C-	866	1.75	-
142	BCN_4	-N-N-N-B-N-C-	894	1.39	-
143	BCN_4	-N-N-B-N-N-C-	896	1.28	-

Supporting Figures

Figure S1 (a)

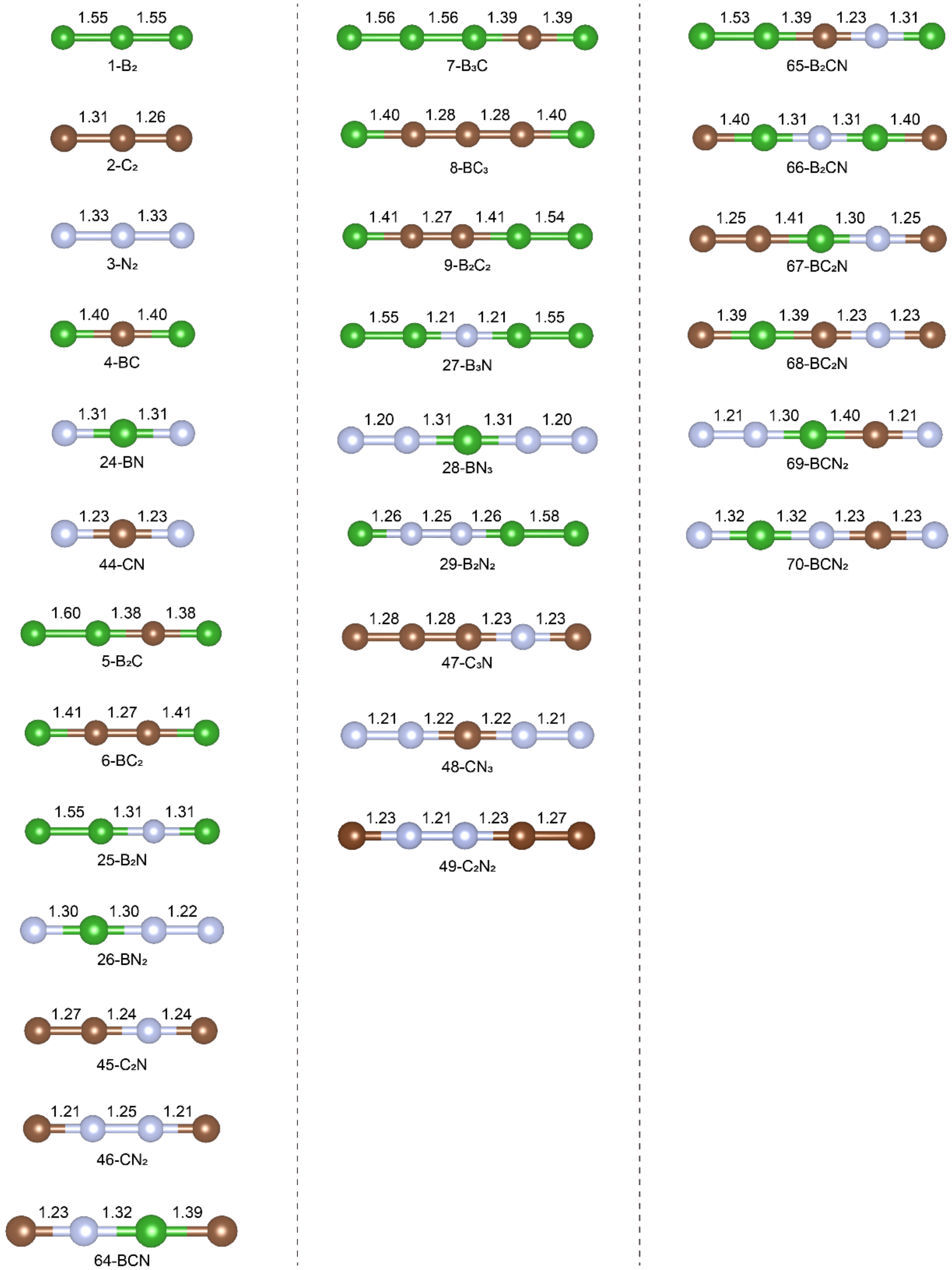

Figure S1 (b)

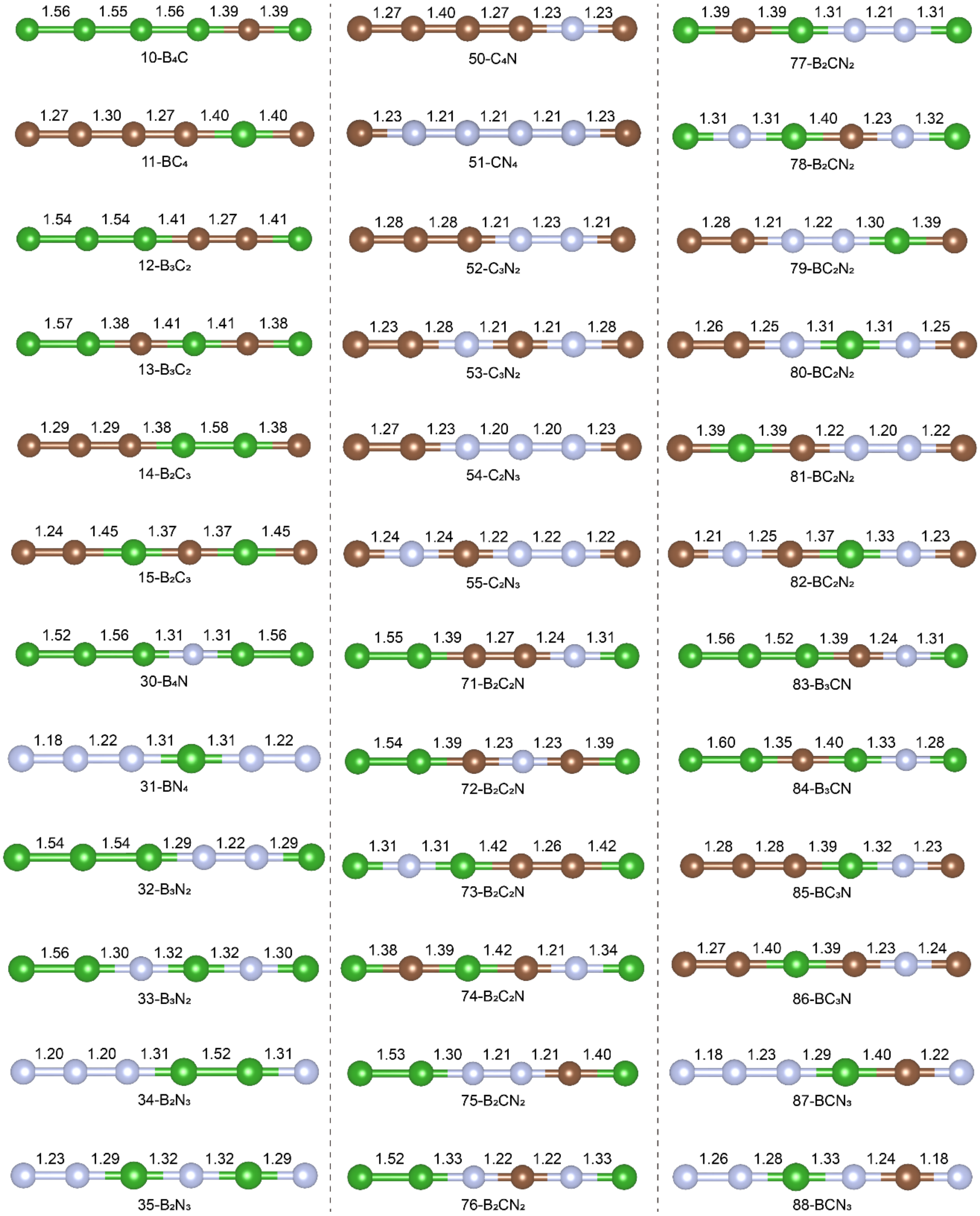
1.56 1.55 1.56 1.39 1.39
10-B_4C
1.27 1.30 1.27 1.40 1.40
11-BC_4
1.54 1.54 1.41 1.27 1.41
12-B_3C_2
1.57 1.38 1.41 1.41 1.38
13-B_3C_2
1.29 1.29 1.38 1.58 1.38
14-B_2C_3
1.24 1.45 1.37 1.37 1.45
15-B_2C_3
1.52 1.56 1.31 1.31 1.56
30-B_4N
1.18 1.22 1.31 1.31 1.22
31-BN_4
1.54 1.54 1.29 1.22 1.29
32-B_3N_2
1.56 1.30 1.32 1.32 1.30
33-B_3N_2
1.20 1.20 1.31 1.52 1.31
34-B_2N_3
1.23 1.29 1.32 1.32 1.29
35-B_2N_3
1.27 1.40 1.27 1.23 1.23
50-C_4N
1.23 1.21 1.21 1.21 1.23
51-CN_4
1.28 1.28 1.21 1.23 1.21
52-C_3N_2
1.23 1.28 1.21 1.21 1.28
53-C_3N_2
1.27 1.23 1.20 1.20 1.23
54-C_2N_3
1.24 1.24 1.22 1.22 1.22
55-C_2N_3
1.55 1.39 1.27 1.24 1.31
71-B_2C_2N
1.54 1.39 1.23 1.23 1.39
72-B_2C_2N
1.31 1.31 1.42 1.26 1.42
73-B_2C_2N
1.38 1.39 1.42 1.21 1.34
74-B_2C_2N
1.53 1.30 1.21 1.21 1.40
75-B_2CN_2
1.52 1.33 1.22 1.22 1.33
76-B_2CN_2
1.39 1.39 1.31 1.21 1.31
77-B_2CN_2
1.31 1.31 1.40 1.23 1.32
78-B_2CN_2
1.28 1.21 1.22 1.30 1.39
79-BC_2N_2
1.26 1.25 1.31 1.31 1.25
80-BC_2N_2
1.39 1.39 1.22 1.20 1.22
81-BC_2N_2
1.21 1.25 1.37 1.33 1.23
82-BC_2N_2
1.56 1.52 1.39 1.24 1.31
83-B_3CN
1.60 1.35 1.40 1.33 1.28
84-B_3CN
1.28 1.28 1.39 1.32 1.23
85-BC_3N
1.27 1.40 1.39 1.23 1.24
86-BC_3N
1.18 1.23 1.29 1.40 1.22
87-BCN_3
1.26 1.28 1.33 1.24 1.18
88-BCN_3

Figure S1 (c)

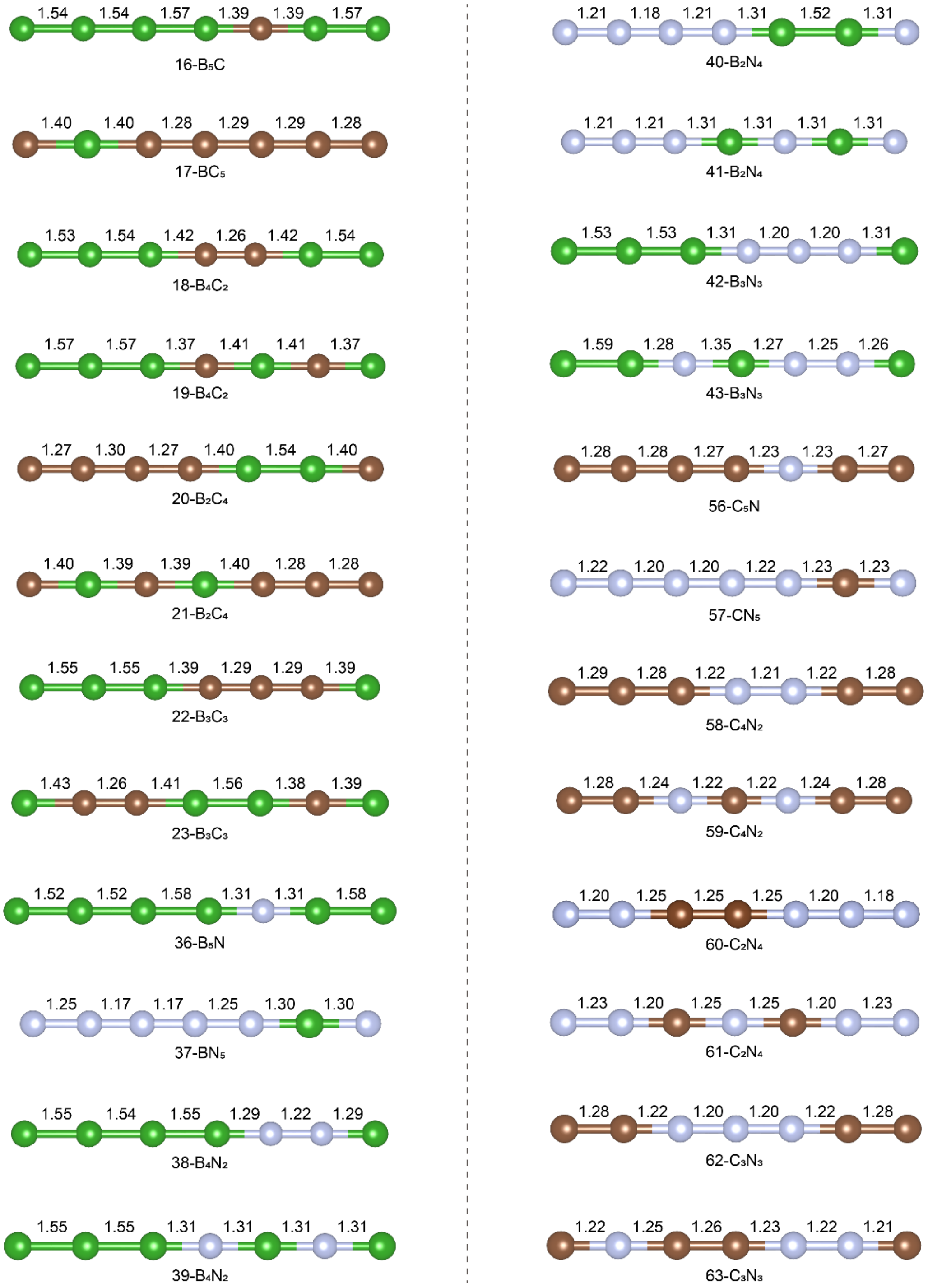
1.54 1.54 1.57 1.39 1.39 1.57
16-B_5C
1.40 1.40 1.28 1.29 1.29 1.28
17-BC_5
1.53 1.54 1.42 1.26 1.42 1.54
18-B_4C_2
1.57 1.57 1.37 1.41 1.41 1.37
19-B_4C_2
1.27 1.30 1.27 1.40 1.54 1.40
20-B_2C_4
1.40 1.39 1.39 1.40 1.28 1.28
21-B_2C_4
1.55 1.55 1.39 1.29 1.29 1.39
22-B_3C_3
1.43 1.26 1.41 1.56 1.38 1.39
23-B_3C_3
1.52 1.52 1.58 1.31 1.31 1.58
36-B_5N
1.25 1.17 1.17 1.25 1.30 1.30
37-BN_5
1.55 1.54 1.55 1.29 1.22 1.29
38-B_4N_2
1.55 1.55 1.31 1.31 1.31 1.31
39-B_4N_2
1.21 1.18 1.21 1.31 1.52 1.31
40-B_2N_4
1.21 1.21 1.31 1.31 1.31 1.31
41-B_2N_4
1.53 1.53 1.31 1.20 1.20 1.31
42-B_3N_3
1.59 1.28 1.35 1.27 1.25 1.26
43-B_3N_3
1.28 1.28 1.27 1.23 1.23 1.27
56-C_5N
1.22 1.20 1.20 1.22 1.23 1.23
57-CN_5
1.29 1.28 1.22 1.21 1.22 1.28
58-C_4N_2
1.28 1.24 1.22 1.22 1.24 1.28
59-C_4N_2
1.20 1.25 1.25 1.25 1.20 1.18
60-C_2N_4
1.23 1.20 1.25 1.25 1.20 1.23
61-C_2N_4
1.28 1.22 1.20 1.20 1.22 1.28
62-C_3N_3
1.22 1.25 1.26 1.23 1.22 1.21
63-C_3N_3

Figure S1 (d)

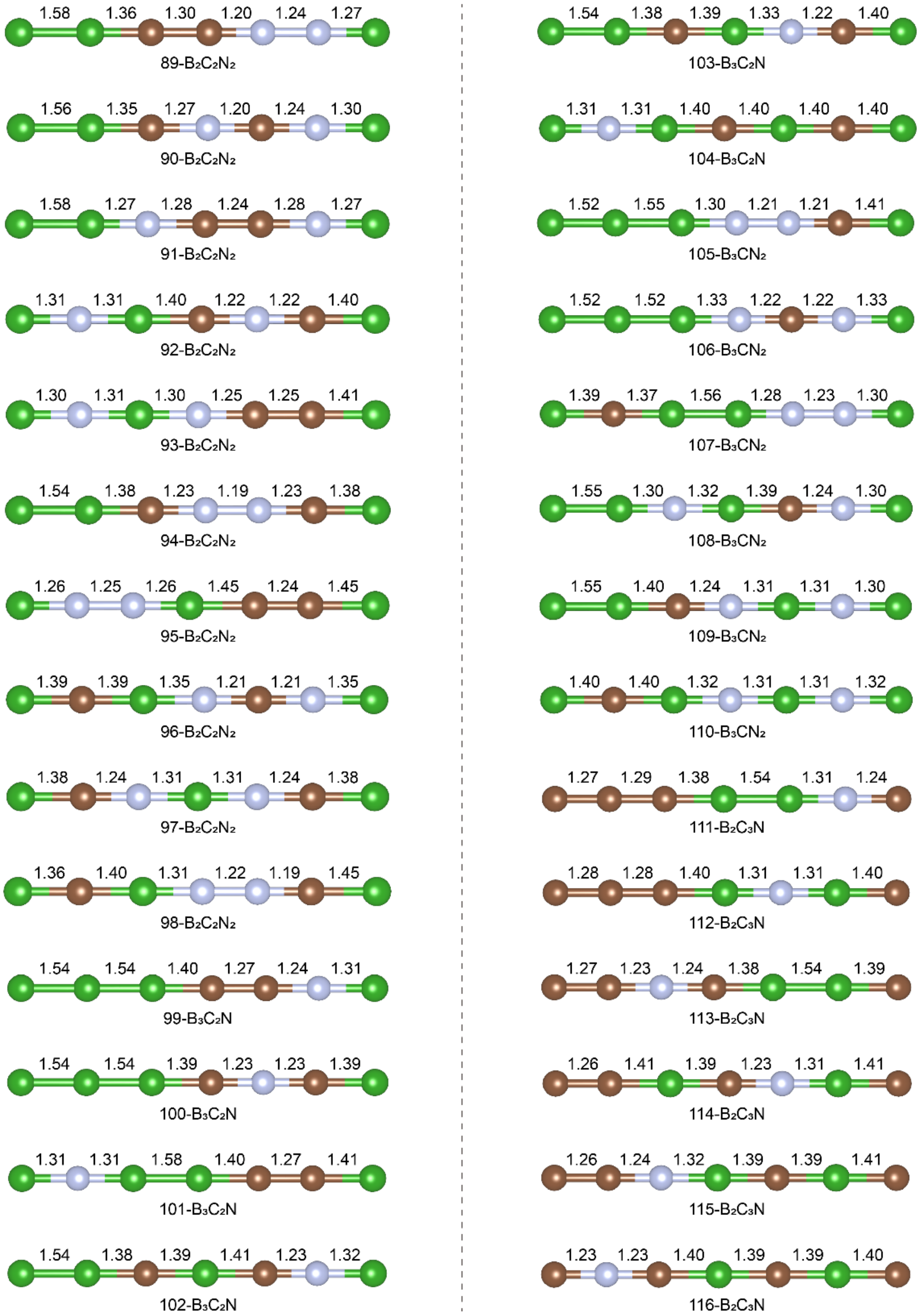
1.58 1.36 1.30 1.20 1.24 1.27
89-$B_2C_2N_2$
1.56 1.35 1.27 1.20 1.24 1.30
90-$B_2C_2N_2$
1.58 1.27 1.28 1.24 1.28 1.27
91-$B_2C_2N_2$
1.31 1.31 1.40 1.22 1.22 1.40
92-$B_2C_2N_2$
1.30 1.31 1.30 1.25 1.25 1.41
93-$B_2C_2N_2$
1.54 1.38 1.23 1.19 1.23 1.38
94-$B_2C_2N_2$
1.26 1.25 1.26 1.45 1.24 1.45
95-$B_2C_2N_2$
1.39 1.39 1.35 1.21 1.21 1.35
96-$B_2C_2N_2$
1.38 1.24 1.31 1.31 1.24 1.38
97-$B_2C_2N_2$
1.36 1.40 1.31 1.22 1.19 1.45
98-$B_2C_2N_2$
1.54 1.54 1.40 1.27 1.24 1.31
99-B_3C_2N
1.54 1.54 1.39 1.23 1.23 1.39
100-B_3C_2N
1.31 1.31 1.58 1.40 1.27 1.41
101-B_3C_2N
1.54 1.38 1.39 1.41 1.23 1.32
102-B_3C_2N
1.54 1.38 1.39 1.33 1.22 1.40
103-B_3C_2N
1.31 1.31 1.40 1.40 1.40 1.40
104-B_3C_2N
1.52 1.55 1.30 1.21 1.21 1.41
105-B_3CN_2
1.52 1.52 1.33 1.22 1.22 1.33
106-B_3CN_2
1.39 1.37 1.56 1.28 1.23 1.30
107-B_3CN_2
1.55 1.30 1.32 1.39 1.24 1.30
108-B_3CN_2
1.55 1.40 1.24 1.31 1.31 1.30
109-B_3CN_2
1.40 1.40 1.32 1.31 1.31 1.32
110-B_3CN_2
1.27 1.29 1.38 1.54 1.31 1.24
111-B_2C_3N
1.28 1.28 1.40 1.31 1.31 1.40
112-B_2C_3N
1.27 1.23 1.24 1.38 1.54 1.39
113-B_2C_3N
1.26 1.41 1.39 1.23 1.31 1.41
114-B_2C_3N
1.26 1.24 1.32 1.39 1.39 1.41
115-B_2C_3N
1.23 1.23 1.40 1.39 1.39 1.40
116-B_2C_3N

Figure S1 (e)

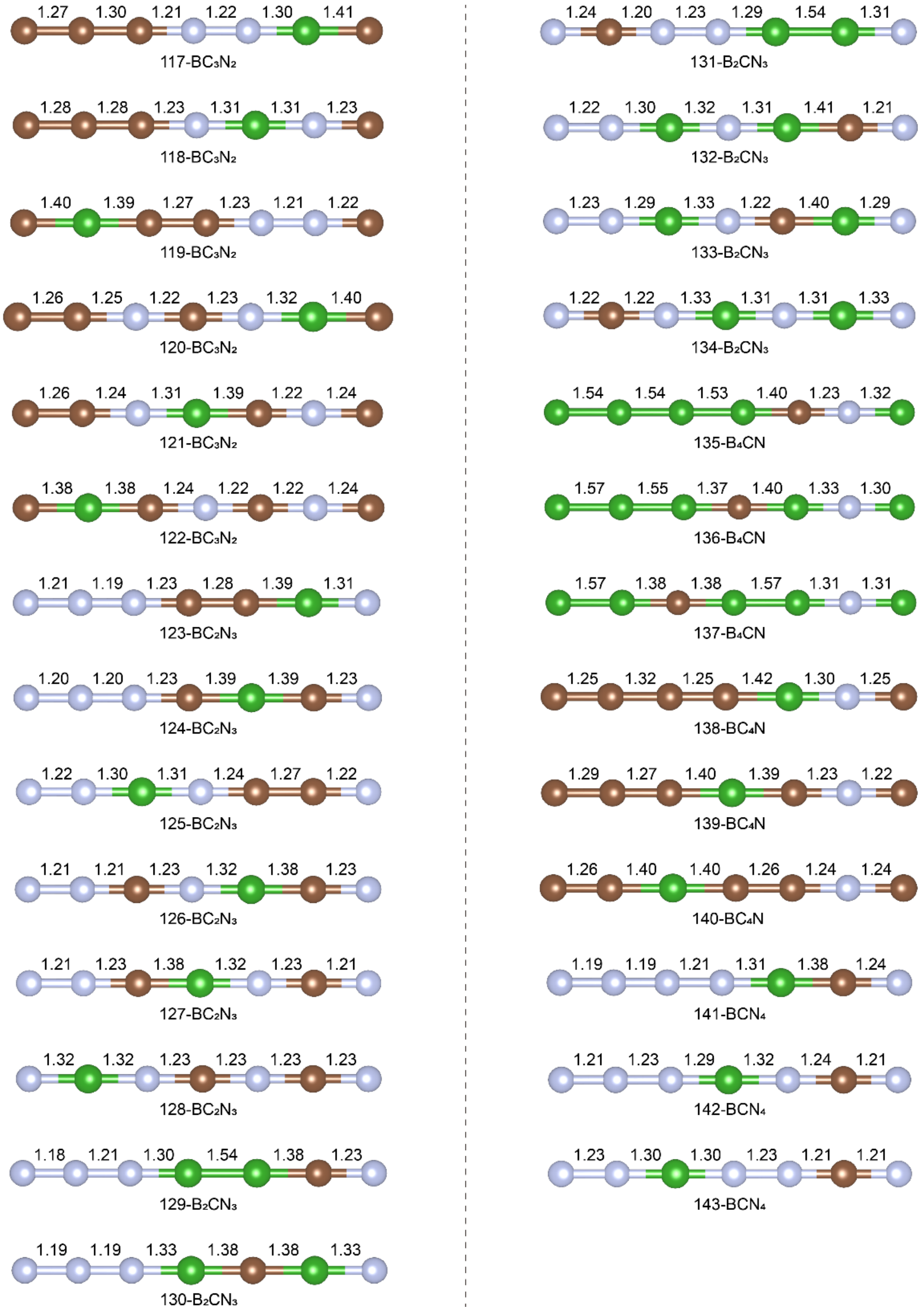

Figure S1. Structures for 143 unique atomic chains of combinations of boron, carbon, and nitrogen. The primitive cells contain a) 4 or fewer, b) 5 and c-e) 6 atoms. The index before the chemical formula corresponds to that in Table S1. The number for each chemical bond indicates the DFT-optimized bond length in units of Angstroms.

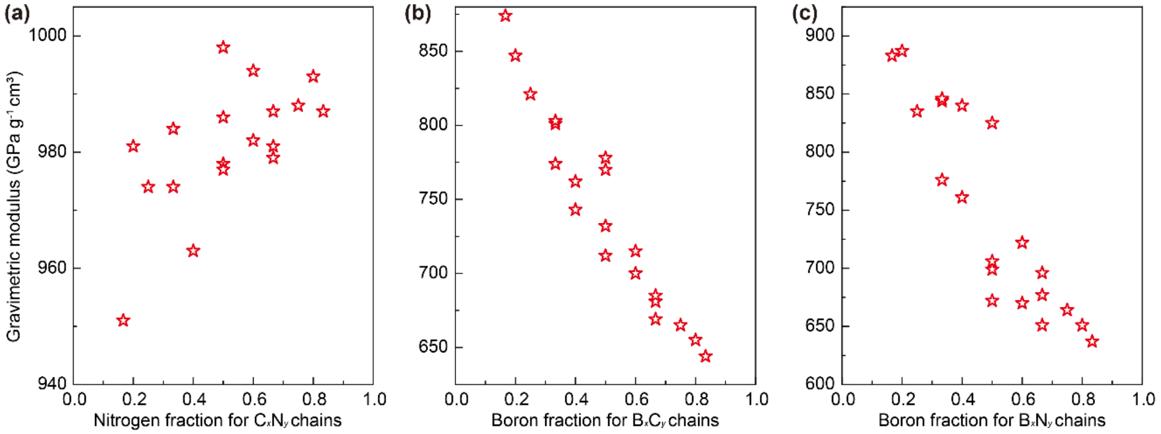

Figure S2. Gravimetric modulus of atomic chains varies with element fractions. a) Gravimetric modulus of C_xN_y chains as a function of nitrogen fraction. b) Gravimetric modulus of B_xC_y chains as a function of boron fraction. c) Gravimetric modulus of B_xN_y chains as a function of boron fraction.

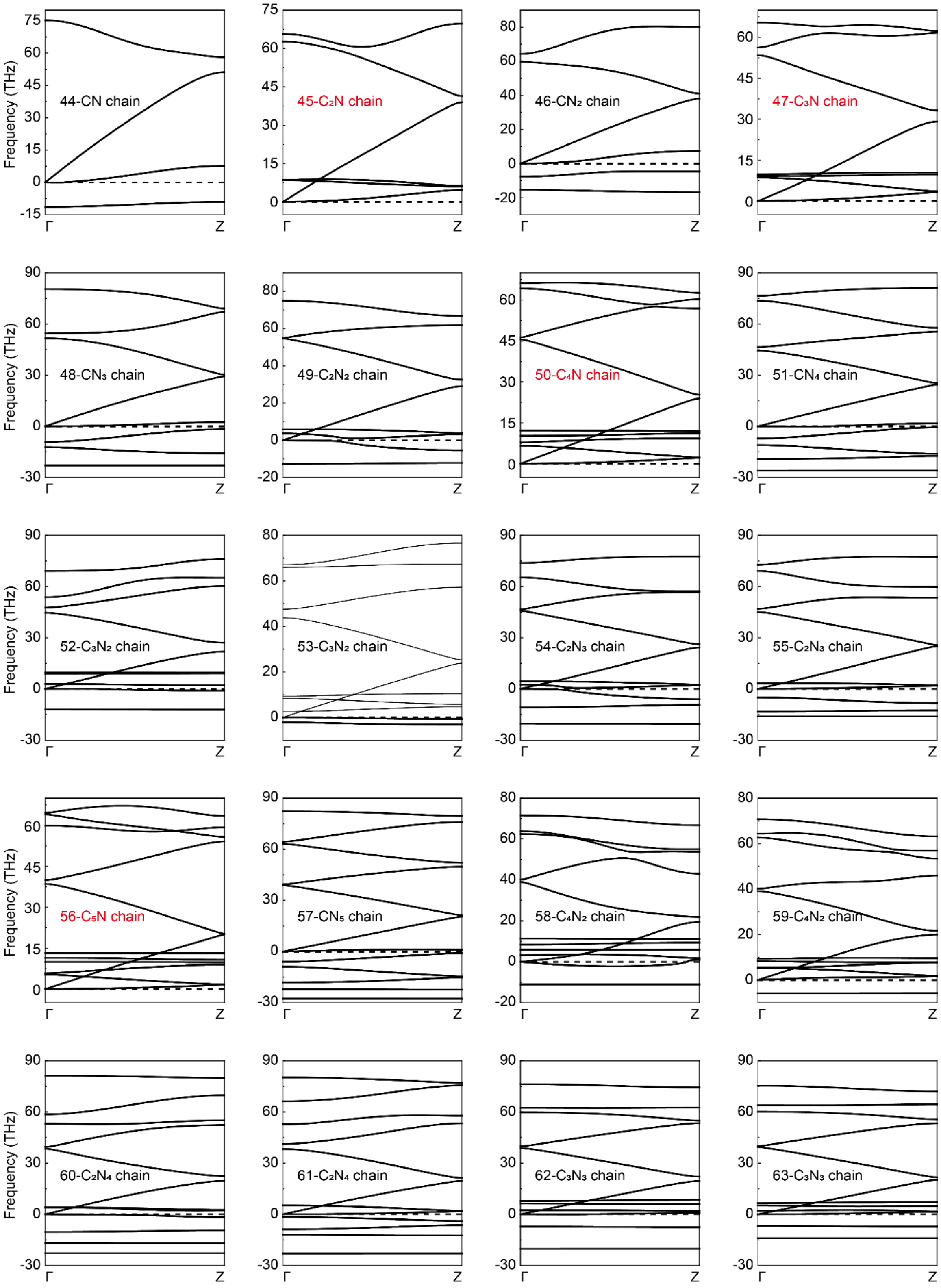

Figure S3. Phonon dispersion curves for C_xN_y chains. The index before the chemical formula corresponds to that in Table S1. The stable chains are labelled with red font.

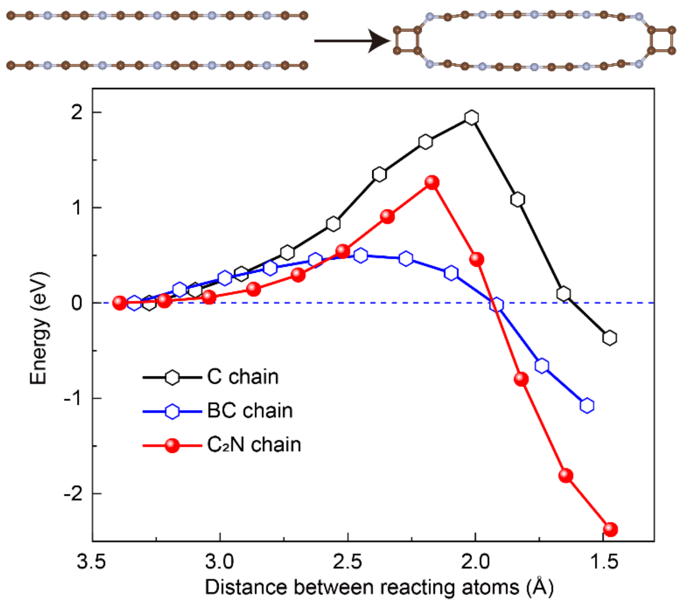

Figure S4. Inter-chain reactions by formation of four-membered rings (top) and the energy change of this reaction per ring as a function of the separation between reacting atoms (bottom). The data for the C and BC chains are from our previous work.[7]

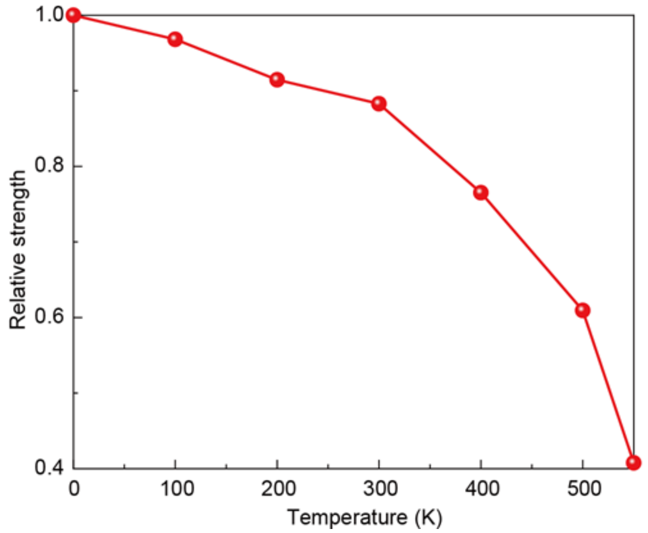

Figure S5. Relative strength for the C_2N chain as a function of temperature.

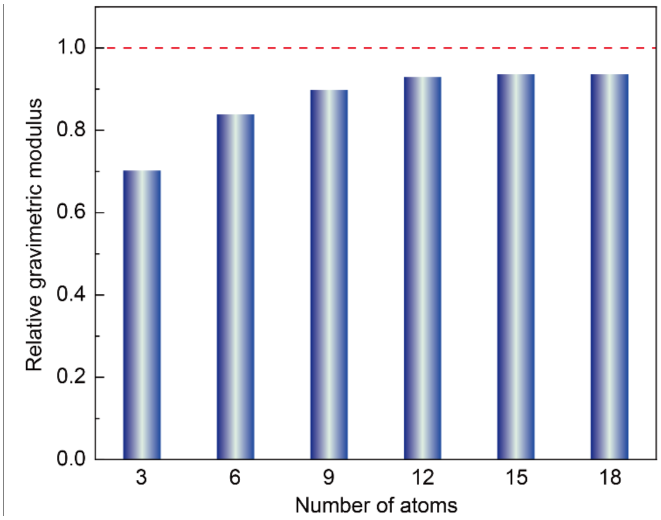

Figure S6. Relative gravimetric moduli (gravimetric moduli divided by the gravimetric modulus for the infinite C_2N chain) for finite C_2N chains as a function of number of atoms.

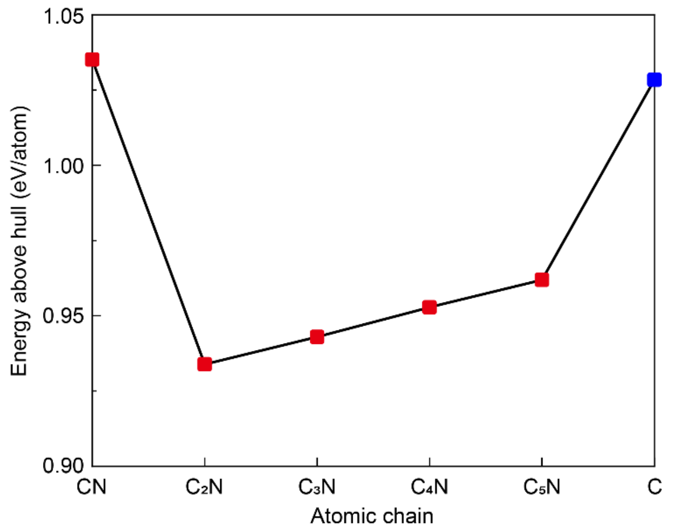

Figure S7. Energies above hull for C_xN chains and the C chain. Note that the CN chain is unstable, as characterized by its response to mechanical perturbations and its phonon dispersion curves.

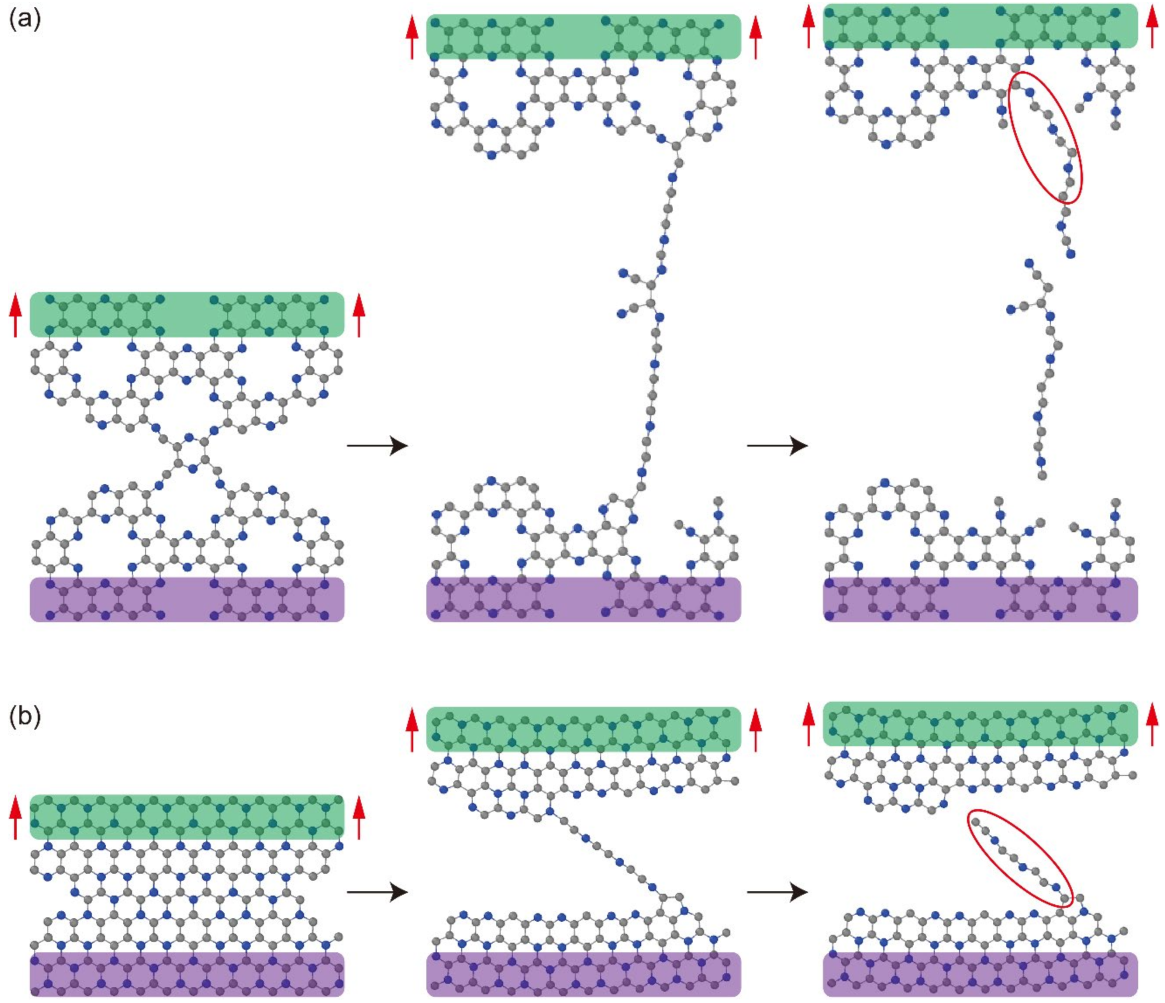

Figure S8. Simulation of the production of C_2N chains by drawing (a) a holey C_2N sheet and (b) a non-holey C_2N sheet. We gradually stretched these structures until rupture by applying strain increments of 1% and minimizing the energy after each increment.

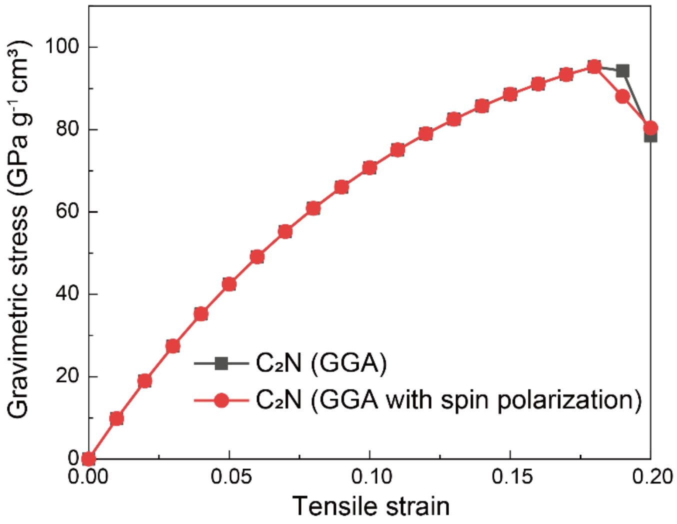

Figure S9. Gravimetric stress-strain curves for the C_2N chain showing the negligible effect of spin polarization in the DFT calculations.

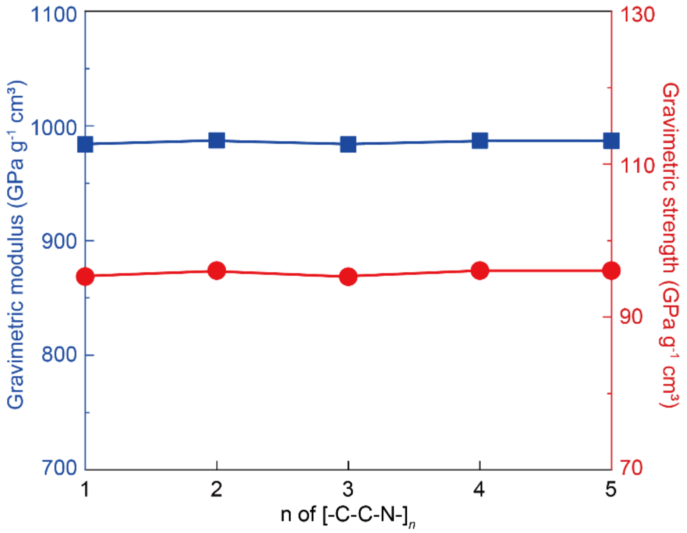

Figure S10. Gravimetric modulus and gravimetric strength for the C_2N chain using different numbers of chain linkages in the computational cell.

Movie S1 (separate file). AIMD simulation movie for the C_2N chains.

Movie S2 (separate file). Simulation of drawing C_2N chains from a holey C_2N sheet.

Movie S3 (separate file). Simulation of drawing C_2N chains from a non-holey C_2N sheet.

Reference

1. R. Puthur, K. L. Sebastian. *Phys. Rev. B* **2002**, *66*, 024304.
2. H. Charan, A. Hansen, H. G. E. Hentschel, I. Procaccia. *Phys. Rev. Lett.* **2021**, *126*, 085501.
3. C. Jin, H. Lan, L. Peng, K. Suenaga, S. Iijima. *Phys. Rev. Lett.* **2009**, *102*, 205501.
4. M. Topsakal, S. Ciraci. *Phys. Rev. B* **2010**, *81*, 205437.
5. B. Hourahine, B. Aradi, V. Blum, F. Bonafe, A. Buccheri, C. Camacho, C. Cevallos, M. Y. Deshaye, T. Dumitrica, A. Dominguez, S. Ehlert, M. Elstner, T. van der Heide, J. Hermann, S. Irle, J. J. Kranz, C. Kohler, T. Kowalczyk, T. Kubar, I. S. Lee, V. Lutsker, R. J. Maurer, S. K. Min, I. Mitchell, C. Negre, T. A. Niehaus, A. M. N. Niklasson, A. J. Page, A. Pecchia, G. Penazzi, M. P. Persson, J. Rezac, C. G. Sanchez, M. Sternberg, M. Stohr, F. Stuckenberg, A. Tkatchenko, V. W. Yu, T. Frauenheim. *J. Chem. Phys.* **2020**, *152*, 124101.
6. M. Gaus, A. Goez, M. Elstner. *J. Chem. Theory Comput.* **2012**, *9*, 338.
7. E. Gao, Y. Guo, Z. Wang, S. O. Nielsen, R. H. Baughman. *Matter* **2022**, *5*, 1192.
